# Chromosomal imbalances associated with anaplastic transformation of follicular thyroid carcinomas

**DOI:** 10.1038/sj.bjc.6601530

**Published:** 2004-01-20

**Authors:** R F Rodrigues, L Roque, J Rosa-Santos, O Cid, J Soares

**Affiliations:** 1 Cytogenetic Laboratory, CIPM, Portuguese Cancer Institute, R. Prof Lima Basto 1099-023, Lisbon, Portugal; 2 Head and Neck Surgery Department, Portuguese Cancer Institute. R. Prof Lima Basto 1099-023, Lisbon, Portugal; 3 Pathology Department, Portuguese Cancer Institute. R. Prof Lima Basto 1099-023 Lisbon, Portugal

**Keywords:** CGH, thyroid, follicular undifferentiation

## Abstract

The genetic alterations that underlie the progression of follicular thyroid carcinoma towards anaplasia are still largely uncharacterised. We compared the Comparative Genomic Hybridization (CGH) profiles of 20 follicular (FTCs), 12 poorly differentiated (PDTCs) and seven anaplastic thyroid carcinomas (ATCs), in order to identify the chromosomal imbalances potentially associated with cancer progression. We found: (i) when considering that a ‘direct’ transformation of FTC towards anaplasia occurs, the defined significantly important alterations were the increase of gains at 3q (*P*<0.05) and 20q (*P*<0.01), and the increase of losses at 7q (*P*<0.05) and Xp (*P*<0.01); (ii) regarding poorly differentiated carcinomas as an intermediate independent entity in the anaplastic transformation of follicular cancers, evidenced as important alterations towards anaplasia, were the proportional decrease in copy sequences at 7p, 7q, 12q and 13q resulting from the significant decrease of DNA gains at 7p and 12q (*P*<0.05), and the significant increase of losses at 7q and 13q (*P*<0.05). These results unveil the chromosomal regions where genes of interest in thyroid anaplastic transformation are to be located, and demonstrate that different gene dosage copy sequence imbalances are associated to the ‘direct’ pathway of transformation of follicular into anaplastic cancers and to the progressive FTC → PDTC → ATC pathway.

Thyroid malignant neoplasms of follicular cell derivation are classified according to the World Health Organization (WHO) criteria into well-differentiated papillary (PTCs) and follicular (FTCs) carcinomas and undifferentiated or anaplastic carcinomas (ATCs) (Hedinger *et al*, 1988). Clinical and pathological data suggest that, in thyroid, PTC and FTC arise through distinct pathways from follicular cells, and that both may dedifferentiate into ATC (Williams, 1979; Wynford-Thomas, 1997). Poorly differentiated thyroid carcinomas (PDTCs) identified by the WHO as a subtype of well-differentiated tumours (WDTCs) show morphological and clinical criteria in between WDTC and ATC, and are identified by some as an intermediate distinct entity between well-differentiated and anaplastic neoplasms (Sobrinho-Simões *et al*, 2002). Genetic studies largely support this model, and it is currently known that activation of *RET*, *NTRK* (Pierotti *et al*, 1996) and *BRAF* (Kimura *et al*, 2003), and overexpression of the *MET* oncogene (Suárez, 1998) contribute to the development of PTC, whereas *RAS* point mutations (Suárez, 1998) and *PPARG/PAX8* rearrangements (Kroll *et al*, 2000) are associated to FTC genesis. The genetic events associated to the progression towards anaplasia are, however, less understood. However, it was identified that the frequency of *P53* (Wynford-Thomas, 1997) and *β-catenin* (Garcia-Rostan *et al*, 1999) mutations raised from WDTC to PDTC to ATC, being significantly higher in the latter. Experiments performed both in *in vitro* as well as in *in vivo* models clearly demonstrated that they could not determine *de per si* anaplastic thyroid transformation, and other genes ought to be involved in that mechanism (Wynford-Thomas, 1997).

CGH analysis represents a useful method to identify the areas of the genome where genes linked to the development and/or progression of neoplasms are to be located. CGH studies in the less-differentiated thyroid forms of cancer are restricted to 15 PDTCs and 38 ATCs (Hemmer *et al*, 1999; Wilkens *et al*, 2000; Wreesmann *et al*, 2002). In PDTC, they were identified as the most frequent changes losses at 11p11–31, 6p21 and 13q21–31. In ATC, different chromosomal regions were identified as relevant. Hemmer *et al* (1999) identified gains of 7p22-pter, 8q22-qter and 9q34-qter as the most frequent imbalances. Wilkens *et al* (2000) defined gains of 5p and alterations of 8 as playing an important role in the development of ATC, whereas, Wreesmann *et al* (2002) proposed that gains at 3p13–14 and 11q13, and loss of 5q11–31, were markers for anaplastic transformation.

In order to further characterise the genomic imbalances associated with the development of anaplastic thyroid carcinomas, we performed a CGH analysis in 12 PDTCs and seven ATCs, and compared the obtained data with the CGH imbalances of 20 previously analysed FTC (Roque *et al*, 2003).

## MATERIAL AND METHODS

### Tumor specimens

In all, 20 FTC, 12 PDTC and seven ATC were included in this study. In which concerns FTC, the median age of the patients was 60 years (range from 33 to 87). The clinical and histological characteristics of this group of patients were those referred in Roque *et al* (2003). Poorly differentiated thyroid carcinoma cases were classified according to the criteria described in the Armed Forces Institute of Pathology (AFIP) (Rosai *et al*, 1992). In the PDTC group, six patients were females and five males. In case 10, two different areas (A and B) from the tumour were analysed (Table 1

**Table 1:** Poorly differentiated thyroid carcinomas (PDTC)–clinical and CGH data

**Case**	**Sex/age**	**CGH results**
1	F/35	+2q23, +4q21–q28, +5p14, +5p11, +5q14–q22, +12q15–q21, +13q21, +14q13–q21
2	F/67	−2q13–qter, +4q26–q28, +7pter–q21, +20, −22q13–qter
3	M/67	+1p21–p10, +1q12–q31, −1q44–qter, −2p16, −2q22–q23, −2q24.3–q34, −3pter–p26, −3p24.1, −3p14.1–p12, −4p15, −4q21.2–q31.2, −4q32–qter, +5pter–q11, +5q31–q34, +6pter–p11, −6q21, +7, −8p23, +8q10–qter, −9pter–p22, +9q22–q32, +9q34, +10q11–qter, −11p15.1−p13, +11q12–qter, +12, −13q21, +13q31–qter, +14q10–qter, +16p13–p11, +17, +18p11.3, +18p11.2–q11.2, +20, −X, −Y
4	F/44	+1q21–q31, +2q11–q37, +3q24–q25, +4q22–q26, +5p15.1–q11*, +6q25, +7p21, +7p15–p13, +7q21–q32, +9pter–p11, +10pter–p11.2*, −10q11.2, −11q21–q22, −11q23.2–qter, +14q24–qter, −16p11.2–p11.1, −Xp14–p11, −Xq28
5	F/75	−1pter–p33, +1p31.1, +1p21, +3p12, −4pter–p13, −4cent, +5p14–p12, +5q11–qter, +6p11–q14, +7, +9pter–q11, +9q12–q22.1, −10q26–qter, −11pter–p15, −11p11–q13, −11q23–qter, −12pter–p13, −12q24–qter, +13q21,+14, −17p13–p12, −17q11–q21, −17q24–qter, +18p11.3, −18p11.1–q21, +18q22–qter, −19, −20p11.2–qter, −21q22.2–qter, −22, +X
6	M/67	+19, −22, −Y
7	M/58	+1pter–p13, −1q12, +1q23–q43, +2p25, +2p22–q35, +3pter–p21, +3q10–qter, +4pter–p14, −4q10–qter, +5, −6, +7pter–p15, +7p14–p11.1, +7q11.2–q32, +8p21–qter, +9pter–p13, +9q10–q13, +10p11.2, +10q11.2–q23, −11, +12pter–q23, +12q24, −13, +14q10–q24, +15q15–q23, +16p11.2, −17, +18p11.3, +18p11–q21, +20pter–p11.2, +20q13.3, +21q10–qter
8	F/79	+1p13, −3p21–p12, +3q22–qter, +5q11.2, −6, +7q11.2, −8pter–p11.2, +9p13, +10, +11q11–q14, −11q22–qter, +12p13, +12p11.2, −13, +14q10–qter, +17p3, +17q21–qter, +18pter–q12, −18q21–qter, +19, +20p11.2, +20q13.1–qter, −X
9	M/50	+1p36.3–p31.3, +1p22–p13.3, +1q12–q32, +1q42–q43, +2p25–p21, +2p14–q11, +2q14.3–q35, +4p14–q11, +4q21, +4q27–q31.2, +6p21.3, +6p21.1–q11, +6q21–q22, −7pter–p21, +7p12–q11.2, +7q22, +8p12–p11, +9p12–q21, +9q22.3, −10q10–qter, +11p11.2, −11q13.5, −11q14–q23, +12q10–q13, +12q21, +13q11–q13, −14q21–qter, +15q22–q24, +16pter–q12.1, −16q12.2–qter, +17p11.2–q24, +18p11.2–p10, +19p13.2–q13.1, +20p10–q12, −22q10–qter, −Y
10 A (Area A)	F/38	+7, +12, +14, +X
10 B (Area B)	F/38	−6p21.3, +7, +9q31, −11q12–q13, +12, +14, −19, −21q22–qter, −22q12–qter, +X
11	M/42	+3p23–p21.3, +3p21.1–q26.3, +5p15.2–q14, +5q33, +9p12–q21, +12p11–q23, +16p11.2

F=female; M=male;

* in bold–regions where high amplifications were observed.

). The median age of patients with PDTC was 57 years (range from 35 to 79). In the ATC group, all patients were females (Table 2

**Table 2:** Anaplastic thyroid carcinomas (ATC)–clinical and CGH data

**Case**	**Sex/age**	**CGH results**
1	F/51	−13
2	F/77	−1pter–p13, +2, −3pter–p14, +3p12.1–qter*, −4q10–qter, +5p13–qter, +6pter–p10, +7pter–q22, −8pter–p23, +8q10–qter, +9pter–p13*, +9p11–q12, +9q33–qter, −10pter–p11.2, +11q12–q13, −11q14.3–q23.2, +12pter–q12*, +16q10–q11.2, +16q21–qter, −17p13.1–p11.2, −18, +19p13.2, +20, −Xpter–p10
3	F/74	+1pter–p13, +3pter–p23, +3p11.2–qter, +4q10–qter, +5, −7q22–q32, +11pter–p12, +13q34–qter, +14q12–qter, +18p11.1–qter*, +19q12–q13.1, +19q13.3, +20, −Xpter–Xq22
4	F/72	+1pter–p36, +1p35, +1p32, +1p31.1–p22, −2q33–qter, +3q26–qter, −4q33–qter, +5pter–p10*, +7pter–p21, −7q31, +9pter–p24, −9p21–p12, −12pter–p11.1, −13q10–q21, +14q10–qter*, +15q21–qter, +19p12.1, +19q13.2, **+20**, −Xpter–p11.1, −Xq10–qter
5	F/66	+1pter–p13, +1q12–q21, +1q25–qter, +2p23–p15, +2q14.1–q24, −3pter–p25, +3q11.2–qter, +4pter–4q31.3, +5pter–q33, +6p23–q15*, +6q22, +7pter–p21, −7q10–qter, +8q10–qter, −9pter–p24, +10pter–p11.2, +11q12, −11q14–qter, −12p12, +12q14–q21.3, +14q10–qter, +15q10–qter, +16q10–qter, −17p12–p11, +17q22–qter, −18p11–qter, +19p13.1–p11.2, +20p11–qter, +21q21.2–qter, −X
6	F/77	−2pter–p24, +2q23–q32, +2q33.2–q35, −3p14–p12, +4p15.2–q32, +5p11–qter, +7q22–q34, −8pter–p12, +8q10–qter, −9pter–p21, +9q22–qter, +10, +12pter–p11, −12q15–qter, −13q12–q31, −15q10–qter, −17pter–p11, −17q11–q22, +20p11.2–qter, +21q21.1–qter, +22q11–qter, −Xpter–p11.2
7	F/64	+1pter–p13, −2q33, +3q27, −4q33–qter, −5q13–q21, +9pter–p22, +9q22–qter, +10q24–qter, +12pter–p13, −13q21–q33, +14q32–qter, +17, +19pter–p10, +20p11–qter, −21q11.2–q21, +22q12–qter, −Xp11.2–q13.

F=female; M=male;

* in bold–regions where high amplifications were observed.

), being their median age 69 years (range from 51 to 77).

### CGH

CGH analysis was performed from frozen material stored in liquid nitrogen at the Cytogenetic laboratory – CIPM of the Lisbon Portuguese Cancer Institute. Microscopic examination of all tumour samples was performed before CGH analysis, in order to be certain that in all cases at least 70% of the cells had the histological characteristics of the group to which that specimen was ascertain. DNA was isolated using a standard phenol-based method (Sambrook and Russel, 2001).

CGH was performed according to the method of Kallioniemi *et al* (1994) and as previously described (Roque *et al*, 2003). Briefly, tumour DNA was labelled with biotin 16dUTP (Enzo-Roche) and normal reference DNA with digoxigenin-11-dUTP (Enzo-Roche), in a standard nick translation reaction. Equal amounts (400 ng) of labelled tumour DNA and labelled reference DNA were co-precipitated with 15 *μ*g of Cot-1-DNA (Invitrogen) in ethanol. After a 3-day hybridisation period, fluorescent detection of the biotin- and digoxigenin-labelled DNAs was accomplished by using avidin–FITC (Jackson Immunoresearch) and antidigoxigenin rhodamine (Enzo-Roche) antibodies, respectively. Samples were counterstained in DAPI in antifade solution (Vector).

For image acquisition, an epifluorescent microscope (Zeiss Axioplan II) equipped with a cooled CCD camera (Photomic Science) and a triple-band beam splitter and emission filters (Chroma Technology, USA) were used. For each tumour, three-colour images (blue, red and green) were acquired from at least 10 metaphases. Image analysis was performed using the CGH analysis software from a CytoVision System (version 2.51 Applied Imaging, Sunderland Tyne & Wear, UK).

Chromosomal regions were interpreted as gained when the red–green profile ratio exceeded 1.25; as highly amplified when the ratio exceeded 1.5; and under-represented when the ratio was less than 0.75. Heterochromatic regions in chromosomes 1, 9, 16 and Y, and the p arms of the acrocentric chromosomes were discarded from the analysis.

### Statistical analysis

For statistical analysis, we used the published CGH data of our 20 FTC (Roque *et al*, 2003). The statistical significance of the differences in CGH imbalances between the three different subgroups of thyroid neoplasms, FTC, PDTC and ATC, were determined using Fisher's exact test using STATA^tm^ 4.0 software. Statistical significance was defined as a two-tailed *P*≤0.05.

## RESULTS

### CGH

**Figure 1:**
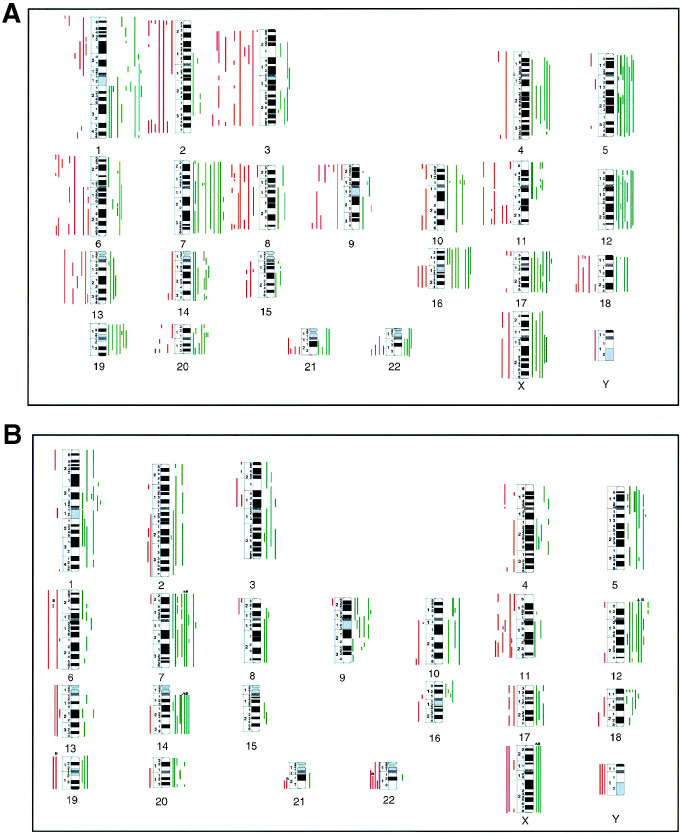
(**A**) Overview of the DNA copy number changes detected by comparative genomic hybridisation in 20 follicular thyroid carcinomas, as previously reported in Roque *et al* (2003). (**B**) Overview of the DNA copy changes observed in the 12 PDTCs. Each line to the left side of the chromosome ideograms represents a loss observed in one tumour, and each line to the right side of the chromosome ideograms represents a gain in one tumour. High-level amplifications are represented by thicker lines.

The chromosomal imbalances observed in FTC were those referred in Roque *et al* (2003). An overview of the observed alterations in FTC is shown in Figure 1A

.

All PDTC presented CGH imbalances (Table 1). Overall, DNA gains were more frequent than losses (Figure 1B). No DNA losses were detected at 3q, 5p/5q, 7q, 9q, 10p, 15q and 20p. Considering only imbalances occurring in at least five tumours (this number was empirically defined as a recurrence threshold), the most frequent changes were gains of 7q occurring, respectively, in 75% (nine out of 12) of the neoplasms, gains of 7p and 14q in 67% (eight out of 12), gains of 12q in 58% (seven out of 12), gains of 1p, 5p/5q, 9p/9q, 12p and loss of 11q in 50% (six out of 12), and gains of 18p and 20q in 42% (five out of 12). Minimal recurring regions of gains were evidenced at 1p21, 3q24–25, 5p14, 7p12 and 7p15–p13, 7q22, 9p13, 12q11–q13, 12q21, 14q13–q21 and 20p12. Minimal regions of losses were defined at 11q23 and 13q21. High-level amplifications were observed in case 4 of this group, involving regions 5p15.1–q11 and 10pter–p11.2.

**Figure 2:**
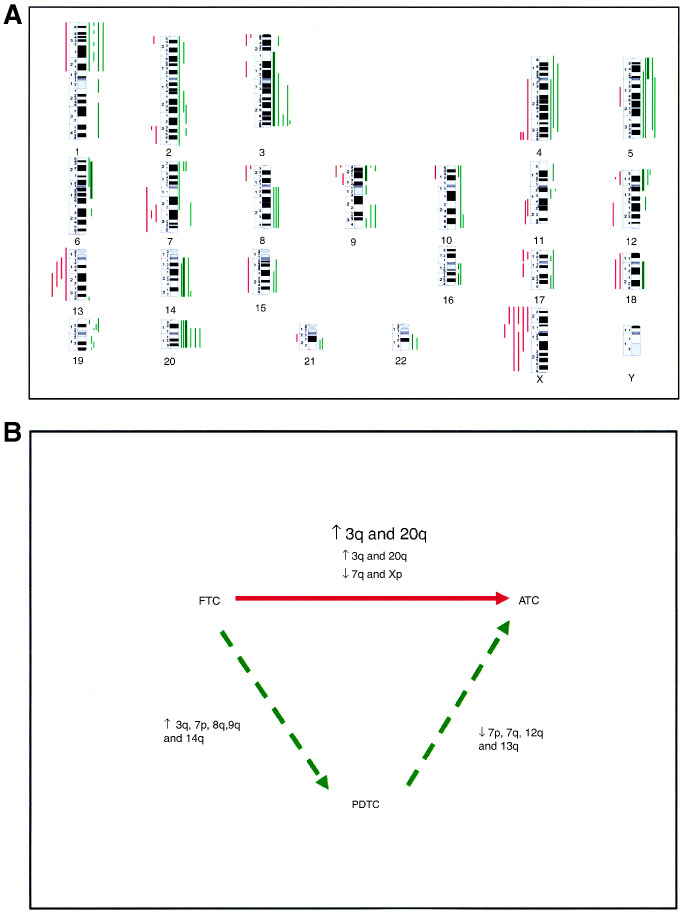
(**A**) Overview of the DNA copy number changes detected by comparative genomic hybridisation in the seven anaplastic thyroid carcinomas. Each line to the left side of the chromosome ideograms represents a loss observed in one tumour, and each bar in the right side of the chromosome ideograms represents a gain in one tumour. High-level amplifications are represented by thicker lines. (**B**) Schematic representation of the CGH copy sequences increases (↑) and copy sequences decreases (↓) defined to be of significance in the two pathways of dedifferentiation of follicular cancers towards anaplasia. FTC=follicular thyroid carcinomas, PDTC=poorly differentiated thyroid carcinomas, ATC=anaplastic thyroid carcinomas.

In the group of anaplastic carcinomas, all cases also presented chromosomal imbalances (Table 2). An overview of the CGH alterations observed in each of the cases is shown in Figure 2A

. Gains were detected in all chromosome arms; however, losses were not observed in a series of regions: 1q, 3q, 4p, 5p, 6p/6q, 8q, 10q, 11p, 14q, 16p/16q, 19p/19q and 20p/20q. In ATC, the number of chromosomal arms affected in imbalances in five or more cases was lower than in the PDTC group. This might be due to the smaller number of tumours evaluated (*n*=7), but it might also reflect a greater interneoplastic genetic heterogeneity. Only four types of chromosomal imbalances were detected as recurrent changes in at least five ATC: gains at 20p/q and losses of Xp in six cases (86%), and gains at 3q and 5p in five of the tumours (71%). At chromosome 7, a minimal region of loss was defined at 7q31. The number of ATC presenting high-level amplifications was higher than in the previous group. High-level amplifications were detected in four of the seven ATC (57%), and different regions were affected in each of the cases. The affected chromosomal regions were 3p12.1-qter, 5pter-p10, 6p23-q15, 9pter-p13, 14q10-qter, 18q 11.1-qter and chromosome 20.

### Statistical analysis

Statistical analysis focused on the genomic differences between the three groups of thyroid neoplasms, considering that in follicular cancerigenesis there is a continuum of dedifferentiation and that those imbalances detected as significantly different would harbour genes involved in neoplastic progression. In the PDTC group, in case 10, the two distinct tumour areas (A and B) were considered as independent samples, since the obtained CGH results, although similar, were not identical. We performed two types of statistical evaluation, one considering that FTC may evolve ‘directly’ to ATC and the other that PDTC represents an intermediate distinct entity in the evolution of FTC towards ATC. In the former analysis, it was identified as statistically significant between the two groups: the increase of gains at regions 3q (*P*<0.05) and 20q (*P*<0.01), and the increase of losses at 7q (*P*<0.05) and Xp (*P*<0.01). Between FTC and PDTC, an increase in the frequencies of gains at: 7p, 9q and 14q and a decrease in the frequencies of losses at 3q and 8q were observed to be significantly different (*P*<0.05). Between PDTC and ATC, the decrease in the frequencies of gains at 7p and 12q, and the increase in the frequencies of losses at regions 7q and 13q, were also statistically significant (*P*<0.05).

## DISCUSSION

Malignant solid tumours are characterised by chromosomal changes that reveal a tumour-specific distribution at early stages of tumorigenesis. The acquisition of such aberrations is a continuous process, and provides a random background of genetic variability in the tumour. It is suggested that certain genetic alterations that emerge in a cancer cell confer that cell a selective advantage over the others, and cancer progression results from the expansion of clone(s) that collectively acquires the following characteristics: self-sufficiency in growth signals, insensitivity to anti-growth signals, evasion to apoptosis, sustained angiosenesis, limitless replicate potential and capacity of tumour invasion and metastasis (Hanahan D and Weinberg RA, 2000).

In thyroid, the chromosomal imbalances associated with anaplastic transformation of follicular thyroid carcinomas are still largely uncharacterised. On the basis that in a significant number of patients anaplastic cancers develop suddenly after a long period of a neoplasm of the differentiated type has been diagnosed, we compared the frequency of chromosomal imbalances in FTC and ATC, as an approach to decipher the genetic factors implicated in this process. Accordingly, we identified that gains of 3q and 20q and losses of 7q and Xp are to play a significant role in committing follicular cancer cells to anaplasia (Figure 2B).

Gains at 3q were observed in five of the seven ATC tumours and a high-level amplification (HLA) was detected in case 2. Several candidate genes are mapped at 3q, which may be deregulated by copy number increase and may be involved in cellular dedifferentiation, for example, *SST*, *PI3K*, *or BCL-6*. *PI3K* is a member of a cell signal pathway that is activated by tyrosine kinases via its regulatory p85 subunit, or by *RAS* via its catalytic p110 subunit (De Vita *et al*, 2000). In thyroid cells, it was reported (Cass *et al*, 1999) that activation of *PI3K* confers a TSH independent DNA synthesis, and it was demonstrated (Gire *et al*., 2000) that in primary human thyroid cells PIK3 is an absolute requirement for the proliferative response to *RAS*. *RAS*-activating point mutations are reported to occur at a high frequency (from 55 to 100%) in undifferentiated thyroid cancers (Suárez, 1998), and thus it is conceivable that an increase in PI3K copy number is implicated in proliferative advantage in these tumours.

A number of genes related to invasion and metastasis (e.g. MMP9 and MMP24), cell cycle and mitotic checkpoints (e.g. *STK15*) are assigned to 20q. Our findings of a significant association (*P*<0.01) between 20q over-representation and anaplasia are in keeping with previous results, which reported that 20q gains were only detected in PDTC and ATC (Wreesmann *et al*, 2002), and with studies performed in thyroid *in vitro* models (Zitzelsberger *et al*, 2001). Zitzelsberger *et al* (2001) detected that 20q gain was an important alteration acquired by thyroid cells in order that they could be able to cause tumours in athymic mice. Interestingly, the authors of this study verified that tumorigenecity in mice required the co-presence of deletions of 9q32–q34 and 7q21–q31, and that varying combinations of these three aberrations could contribute to a more aggressive behaviour.

In our series, we also identified a statistically significant association (*P*<0.05) between 7q losses and ATC. The common deleted region to all cases with 7q loss in this group of tumours was 7q31. The second most common aphidicolin-inducible fragile site of the human genome (Fra7G) has been located in this region, and loss of heterozygosity (LOH) studies indicate this chromosomal region as a frequent site of allele loss in a variety of tumours (Zenklusen *et al*, 2000). In the thyroid gland, it was proposed (Trovato *et al*, 1999) that 7q31 was the site of at least one suppressor gene involved in follicular tumorigenesis.

Six of the seven anaplastic tumours of this series presented deletions at the short arm of the X-chromosome, and a highly significant association (*P*<0.001) was detected between Xp sequence losses and ATC. An association between Xp loss and undifferentiated thyroid cancers has not been previously reported by CGH or by any other technique (Ward *et al*, 1998; Kitamura *et al*, 2001). The DNA losses detected by CGH in our cases were extensive, and we were not able to define minimal chromosomal region(s) of deletions, which may be explained by the limitations of the CGH technique. Alternatively, we may consider that under-representation of a series of genes at Xp may interact or modify the expression of other genes, and therefore promote the progression towards anaplasia. Wynford-Thomas (1997) proposed, based on the use of viral oncogenes (SV40) to mimic the effect of tumour-suppressor gene function loss, that one of the cooperating events of *P53* in thyroid anaplastic cancer could be of epigenetic nature, rather than mutational. Our findings of extensive Xp loss with a highly significant association with ATC make this an attractive hypothesis to explore.

Poorly differentiated thyroid carcinomas consist in a morphological and biologically heterogeneous group of tumours that have been proposed to represent an intermediate independent entity in between WDTC and ATC, and not only a subtype of WDTC (Sobrinho-Simões *et al*, 2002). In keeping with the above proposal, we compared our data in order to determine the genetic imbalances with a significant importance in this progression pathway. Accordingly, we identify that a proportional increase in copy sequences at 3q, 7p, 8q, 9q and 14q, resulting from the significant increase of DNA gains at 7p, 9q and 14q, and the significant decrease of DNA losses at 3q and 8q, would commit the follicular cancer cell to a poorly differentiated phenotype (Figure 2B). CGH data on PDTC are scarce and we are aware of only a report by Wreesmann *et al* (2002) describing chromosomal imbalances in 15 PDTC. At variance with our results, they are found to be present in PDTC but not in well-differentiated cancers: gains at 1p34–36, 6p21, 9q34, 17q25 and 20q, and losses at 11p11–31, 2q23–33, 4q11–13, 6q21 and 13q21–31. Interestingly, the series of WDTC analysed by Wreesmann *et al* (2002) was only composed of papillary tumours, a group of differentiated thyroid cancers which is characterised by molecular and chromosomal aberrations distinct from those that were ascertained to FTC development.

From the data obtained in this study, evidenced as important in the progression of PDTC towards anaplasia, were the proportional decrease in copy sequences at 7p, 7q, 12q and 13q, resulting from the significant decrease of DNA gains at 7p and 12q, and from the significant increase of losses at 7q and 13q (Figure 2B). Chromosomal imbalances defined to be of relevance in this pathway of transformation were not identical to those previously referred to be of importance in the ‘direct’ evolution of FTC to ATC. These results are conceivable, taking in account the model that cancer progression is a dynamic process and that in cells with distinct genetic backgrounds the aberrations that continuously emerge and ensure a more aggressive phenotype may not be identical in the different cells. Overall, in the pathway of evolution of PDTC to ATC, loss of sequences seems to represent critical events, namely those observed at chromosomes 7 and 13. Loss of heterozygosity at 13q is a common event in a variety of endocrine tumours, suggesting the involvement of oncosuppressor genes mapped at this chromosomal arm in the pathogenesis of a broad number of endocrine neoplasms. In thyroid neoplasms, LOH studies have only focus at the 13q14 region, and an LOH rate of ≅20% was reported in both benign and malignant tumours (Zedenius *et al*, 1996; Kitamura *et al*, 2001). In our CGH study, DNA losses were observed in all groups of neoplasms. However, whereas in follicular carcinomas, we have defined two minimal regions of losses: one at 13q12–14 and other at 13q32–qter (Roque *et al*, 2003), in PDTC we identified 13q21 as the minimal region of loss. In ATC, large areas of deletions were observed, always including loss at 13q21. Our results suggest that genes involved in dedifferentiation are probably located at 13q21, being distinct from those involved in the pathogenesis of FTC. Of note is that Wreesmann *et al* (2002) in their CGH analysis reported that deletions at 13q21–q31 were exclusively observed in PDTC and ATC.

In conclusion, this study revealed two distinct pathways of genomic imbalances in anaplastic transformation of follicular thyroid carcinomas: one assigned with a ‘direct’ dedifferentiation of FTC into ATC and the other associated with the existence of an intermediate morphologic entity, the PDTC. This inventory of genetic aberrations increases our knowledge of FTC progression and provides useful indications towards the identification of the genes involved.

## References

[bib1] Cass LA, Summers SA, Pendergast GV, Backer JM, Birnbaum MJ, Meinkoth JL (1999) Protein kinase A-dependent and -independent signaling pathways contribute to cyclic AMP-stimulated proliferation. Mol Cell Biol 19: 5882–5891 10454535 10.1128/mcb.19.9.5882 PMC84437

[bib2] De Vita G, Berlingieri MT, Visconti R, Castellone MD, Viglietto G, Balssarre G, Zannini M, Bellacosa A, Tsichlis PN, Fusco A, Santoro M (2000) Akt/protein kinase B promotes survival and hormone-independent proliferation of thyroid cells in the absence of dedifferentiating and transforming effects. Cancer Res 60: 3916–3920 10919669

[bib3] Garcia-Rostan G, Tallini G, Herrero A, D'Aquila TG, Carcangiu ML, Rimm DL (1999) Frequent mutation and nuclear localization of *β*-catenin in anaplastic thyroid carcinoma. Cancer Res 59: 1811–1815 10213482

[bib4] Gire V, Marshall C, Wynford-Thomas D (2000) PI-3-kinase is an essensial anti-apoptotic effector in the proliferative response of primary human epithelial cells to mutant RAS. Oncogene 19: 2269–2276 10822377 10.1038/sj.onc.1203544

[bib27] Hanahan D, Weinberg RA (2000) The hallmarks of cancer. Cell 100: 57–70 10647931 10.1016/s0092-8674(00)81683-9

[bib5] Hedinger CE, Williams ED, Sobin L (1988) Histological typing of thyroid tumours. World Health Organization. International Histological Classification of Tumours, 2nd ed., Berlin: Springer-Verlag

[bib6] Hemmer S, Wasenius VM, Knuutila S, Franssila K, Joensuu, H (1999) DNA copy number changes in thyroid carcinoma. Am J Pathol 154: 1539–1547 10329606 10.1016/S0002-9440(10)65407-7 PMC1866579

[bib7] Kallioniemi O, Kallioniemi A, Piper J, Isolda J, Waldman FM, Pinkel D (1994) Optimizing comparative genomic hybridization for analysis of DNA sequence copy number changes in solid tumours. Genes Chromosom Cancer 10: 231–243 7522536 10.1002/gcc.2870100403

[bib8] Kimura ET, Nikiforova MN, Zhu Z, Knauf JA, Nikiforov YE, Fagin J (2003) High prevalence of BRAF mutations in thyroid cancer: genetic evidence for constitutive activation of the RET/PTC-RAS-BRAF signaling pathway in papillary thyroid carcinoma. Cancer Res 63: 1454–1457 12670889

[bib9] Kitamura Y, Shimizu K, Ito K, Tanaka S, Emi M (2001) Allelotyping of follicular thyroid carcinoma: frequent allelic losses in chromosome arms 7q, 11p, and 22q. J Clin Endocrinol Metab 86: 4268–4272 11549660 10.1210/jcem.86.9.7853

[bib10] Kroll TG, Sarraf P, Pecciarini L, Chen CJ, Mueller E, Spiegelman BM, Fletcher JA (2000) PAX8-PPARG1 fusion oncogene in human thyroid carcinoma. Science 289: 1357–1360 10958784 10.1126/science.289.5483.1357

[bib11] Pierotti MA, Bongarzone I, Borello MG, Greco A, Pilotti S, Sozzi G (1996) Cytogenetics and molecular genetics of carcinomas arising from thyroid follicular cells. Genes Chromosom Cancer 16: 1–14 9162191 10.1002/(SICI)1098-2264(199605)16:1<1::AID-GCC1>3.0.CO;2-4

[bib12] Roque L, Rodrigues R, Pinto A, Moura-Nunes V, Scares J (2003) Chromosome imbalances in thyroid follicular neoplasms: A comparison between follicular adenomas and carcinomas. Genes Chromosom Cancer 36: 292–302 12557229 10.1002/gcc.10146

[bib13] Rosai J, Carcangiu ML, De Lellis R (1992) Tumours of the thyroid gland. Atlas of tumor pathology 3rd series. Washington: Armed forces Institute of Pathology

[bib14] Sambrook J, Russel DW (2001) Molecular cloning a Laboratory Manual, Chapter 6.4–6.12., NY: Cold Spring Harbor Laboratory

[bib15] Sobrinho-Simões M, Sambade C, Fonseca E, Soares P (2002) Poorly differentiated carcinomas of the thyroid gland. J Surg Pathol 10: 123–131 10.1177/106689690201000205 12075405

[bib16] Suárez HG (1998) Genetic alterations in human epithelial thyroid tumours. Clin Endocrinol 48: 531–546 10.1046/j.1365-2265.1998.00443.x 9666864

[bib17] Trovato M, Fraggetta F, Villari D, Batolo D, Mackey K, Trimarchi F, Benvenga S (1999) Loss of heterozygosity of the long arm of chromosome 7 in follicular and anaplastic thyroid cancer, but not in papillary thyroid cancer. J Clin Endocrinol Metab 84: 3235–3240 10487693 10.1210/jcem.84.9.5986

[bib18] Ward LS, Brenta G, Mededovic M, Fagin JA (1998) Studies of allelic loss in thyroid tumours reveal major differences in chromosomal instability between papillary and follicular carcinomas. J Clin Endocrinol Metab 83: 525–530 9467569 10.1210/jcem.83.2.4550

[bib19] Wilkens L, Benten D, Tchinda J, Brabant G, Potter E, Dralle H, von Wasielewski R (2000) Aberrations of chromosome 5 and 8 as recurrent cytogenetic events in anaplastic carcinoma of the thyroid as detected by fluorescence *in situ* hybridization and comparative genomic hybridization. Virchows Arch 436: 312–318 10834532 10.1007/s004280050452

[bib20] Williams ED (1979) The aetiology of thyroid tumours. Clin Endocrinol Metab 8: 193–207 428139 10.1016/s0300-595x(79)80017-1

[bib21] Wreesmann VB, Ghossein RA, Patel SG, Harris CP, Schnaser EA, Shaha AR, Tuttle RM, Shah JP, Rao PH, Singh B (2002) Genome-wide appraisal of thyroid cancer progression. Am J Pathol 161: 1549–1556 12414503 10.1016/S0002-9440(10)64433-1 PMC1850764

[bib22] Wynford-Thomas D (1997) Origin and progression of thyroid epithelial tumours: cellular and molecular mechanisms. Horm Res 47: 145–157 9167946 10.1159/000185458

[bib23] Yasui K, Imoto I, Fokuda Y, Pimkhaokham A, Yang Z, Naruto T, Shimada Y, Nakamura Y, Inazawa J (2001) Identification of target genes whithin an amplicon at 14q12–q13 in esophageal squamous cell carcinoma. Genes Chromosom Cancer 32: 112–118 11550278 10.1002/gcc.1172

[bib24] Zedenius J, Wallis G, Svensson A, Bovée J, Hoog A, Backdahl M, Larsson C (1996) Deletions of the long arm of chromosome 10 in progression of follicular thyroid tumours. Hum Genet 97: 299–303 8786068 10.1007/BF02185758

[bib25] Zenklusen JC, Hodges LC, La Cava M, Green ED, Conti CJ (2000) Definitive functional evidence for a tumor suppressor gene on human chromosome 7q31.1 neighboring the Fra 7G. Oncogene 19: 1729–1733 10763831 10.1038/sj.onc.1203488

[bib26] Zitzelsberger H, Bruch J, Smida J, Hieber L, Peddie CM, Bryant PE, Riches AC, Fung J, Weier HU, Bauchinger M (2001) Clonal chromosomal aberrations in simian virus 40-transfected human thyroid cells and in derived tumours developed after *in vitro* irradiation. Int J Cancer 20: 96 (3): 166-77. 10.1002/ijc.1015 11410885

